# The Effect of Lifestyle Intervention on Diabetes Prevention by Ethnicity: A Systematic Review of Intervention Characteristics Using the TIDieR Framework

**DOI:** 10.3390/nu13114118

**Published:** 2021-11-17

**Authors:** Mingling Chen, Gebresilasea Gendisha Ukke, Lisa J. Moran, Surbhi Sood, Christie J. Bennett, Mahnaz Bahri Khomami, Pilvikki Absetz, Helena Teede, Cheryce L. Harrison, Siew Lim

**Affiliations:** 1 Monash Centre for Health Research and Implementation, School of Public Health and Preventive Medicine, Monash University, Clayton, VIC 3168, Australia; mingling.chen@monash.edu (M.C.); gebretecno@gmail.com (G.G.U.); Lisa.Moran@monash.edu (L.J.M.); sood.surbhi@icloud.com (S.S.); mahnaz.bahri-khomami@monash.edu (M.B.K.); helena.teede@monash.edu (H.T.); Cheryce.Harrison@monash.edu (C.L.H.); 2 Department of Nutrition, Dietetics and Food, School of Clinical Sciences, Monash University, Notting Hill, VIC 3168, Australia; Christie.Bennett@monash.edu; 3 Faculty of Social Sciences, Tampere University, 33014 Tampere, Finland; pilvikki.absetz@gmail.com; 4 Diabetes and Endocrine Unit, Monash Health, Clayton, VIC 3168, Australia

**Keywords:** ethnicity, lifestyle intervention, systematic review, type 2 diabetes

## Abstract

Lifestyle intervention is effective in preventing type 2 diabetes mellitus (T2DM), but the efficacy of intervention components across different ethnic groups is less clear. This systematic review examined the effects of intervention characteristics of lifestyle interventions on diabetes incidence and weight loss by ethnicity using the Template for Intervention Description and Replication (TIDieR) framework. MEDLINE, EMBASE and other databases were searched for randomized and non-randomized controlled trials on lifestyle interventions (diet and/or physical activity) in adults at risk of T2DM. Ethnicity was categorized into European, South Asian, East and Southeast Asian, Middle Eastern, Latin American and African groups. Forty-five studies (18,789 participants) were included in the systematic review and 41 studies in meta-analysis. Meta-analysis showed a high number of intervention sessions was significantly associated with a greater reduction in diabetes incidence (*P* = 0.043) and weight (*P* = 0.015), while other intervention characteristics including intervention provider and delivery format did not alter the outcomes (all *P* > 0.05). Additionally, narrative synthesis showed long-term interventions (≥12 months) were associated with significant diabetes risk reduction for all ethnic groups, while short-term interventions (<12 months) were more effective in weight loss in most ethnic groups. There may be ethnic preferences for the optimal number of intervention sessions.

## 1. Introduction

Type 2 diabetes mellitus (T2DM) is a major global health concern [1]. The prevalence of T2DM varies widely by ethnicity [2]. For example, in the US, American Indians/Alaska Natives have the highest prevalence of diagnosed diabetes (14.7%), followed by Hispanic (12.5%), African (11.7%), Asian (9.2%) and white Americans (7.5%) [3]. Lifestyle modification has been demonstrated to prevent T2DM by up to 58% in high-risk individuals through increased physical activity, healthy diet, and weight management [4,5,6,7]. Adaptation and translation of lifestyle interventions for broad population benefit are underway around the world in an attempt to address the diabetes epidemic. A recent meta-analysis showed lifestyle interventions had similar effects in reducing T2DM incidence across ethnic groups, but ethnic differences were found in weight loss achieved [8]. Ethnicity is a social construct that refers to the cultural identity of a group of people including languages, customs and religions [9]. Effective engagement strategies may differ across ethnic groups, necessitating cultural adaptations of diabetes prevention programs [10,11]. Identification of the determinants of intervention success is required to inform implementation and practice [12]. While effective intervention characteristics have been identified for T2DM prevention in the general population, including number of sessions delivered, intervention duration and adherence to guidelines [13,14,15,16,17], these have not been systematically investigated across various ethnic groups. Understanding the optimal intervention strategies within the specific ethnic groups is imperative to guide cultural adaptations and future scale-up of diabetes prevention programs.

Intervention characteristics can be systematically described using the Template for Intervention Description and Replication (TIDieR) framework, which was originally developed to improve the reporting of intervention studies [18]. Therefore, this systematic review aimed to examine the intervention characteristics of lifestyle interventions associated with T2DM prevention in different ethnic groups using the TIDieR framework.

## 2. Materials and Methods

This is a secondary analysis of our recent systematic review on ethnic differences in lifestyle intervention effects on T2DM prevention in adults [8]. The protocol was prospectively registered on PROSPERO (No. CRD42020193503). A comprehensive search was conducted in MEDLINE, EMBASE, Pubmed, CINAHL, PsycInfo, Cochrane Central Register of Controlled Trials, EBM Reviews and the International Clinical Trials Registry Platform with no language restriction for articles published up to June 2020. The search strategy has been previously described [8]. Inclusion criteria were randomized controlled trials (RCTs) and non-randomized controlled trials (non-RCTs) that compared lifestyle intervention/s (diet, physical activity or both) with a control group (usual care, placebo, no intervention or minimal intervention) on T2DM prevention; participants aged ≥ 18 years and at risk of T2DM (e.g., prediabetes, overweight or obesity, history of gestational diabetes, elevated diabetes risk score); and reporting at least one of the following outcomes by ethnicity: diabetes incidence (defined as fasting glucose ≥ 7.0 mmol/L, and/or 2-h glucose ≥ 11.1 mmol/L, and/or HbA_1c_ ≥ 6.5%, or clinical diagnosis by a physician), fasting glucose, 2-h glucose, HbA_1c_, body weight, body mass index (BMI), waist circumference, physical activity, energy intake, energy from fat and fibre intake. All editorials, letters, commentaries, protocols, conference abstracts, dissertations and reviews were excluded.

The primary outcomes of this analysis were diabetes risk reduction (absolute or relative risk reduction) and weight loss (mean or median loss) measured at the end of the intervention. Intervention characteristics were extracted according to the TIDieR checklist [18] including why (theoretical framework), what (intervention type), who (intervention provider), how (delivery format, use of technology), where (intervention location), when (intervention duration), how much (number of intervention sessions), tailoring (i.e., personalized intervention) and how well (fidelity). Table S1 shows the coding of the TIDieR components. Intervention duration was defined as short (<12 months) or long (≥12 months). The total number of intervention sessions was classified as low (≤15 sessions) or high (≥16 sessions) according to the median. Ethnicity was extracted as described in the included studies and subsequently categorized into European, South Asian, East and Southeast Asian, Middle Eastern, Latin American and African groups based on the World Bank regions [19]. No eligible studies on Indigenous populations were identified. The risk of bias of RCTs and non-RCTs was assessed using the Revised Cochrane Risk of Bias tool for Randomized Trials (RoB 2) [20] and the Risk of Bias in Non-randomized Studies of Interventions (ROBINS-I) tool [21], respectively. Two reviewers independently screened the articles (M.C., G.G.U., S.S. and C.J.B.), extracted the data (M.C., G.G.U., S.S. and M.B.K.) and assessed study quality (M.C. and C.J.B.). Any discrepancies were resolved by discussion or arbitration with a third reviewer (S.L.).

Risk ratios (RRs) for diabetes incidence and mean differences (MDs) for body weight were pooled using random-effects meta-analysis. Subgroup analyses by TIDieR components were conducted to assess the effect of intervention characteristics on the outcomes. Due to insufficient studies for meta-analysis, the associations between intervention characteristics and the outcomes by ethnicity were summarized narratively. Publication bias was assessed with funnel plots and Egger’s tests. Analyses were performed using R version 4.0.3 (Free Software Foundation, Inc. 1991, 1999, Boston, MA, USA).

## 3. Results

### 3.1. Identified Studies

From 17,374 articles, 62 articles representing 45 studies met the inclusion criteria. Of these, 41 studies with complete outcome data were included in meta-analyses (Figure S1). The 45 included studies enrolled 18,789 participants (53% female, mean age 32.8 to 63.9 years) across 14 countries. Most of the studies included participants from the European group (*n* = 12), followed by South Asian (*n* = 10), East and Southeast Asian (*n* = 10), Middle Eastern (*n* = 4), Latin American (*n* = 3) and African groups (*n* = 2). Four studies included more than one ethnic group [4,22,23,24].

### 3.2. Intervention Characteristics

The intervention characteristics according to the TIDieR checklist are summarized in Table S2. Thirty-four studies provided a theoretical framework for the intervention, mostly the social cognitive theory, the transtheoretical model and the health action process approach. The remaining studies did not report the theory used to underpin the intervention. All studies utilized a combined diet and physical activity intervention, with the exception of one, diet only intervention [25]. Thirty-three studies involved health professionals as the intervention provider (e.g., dietitian, nurse, physician, physiotherapist, healthcare worker), while interventions in the rest studies were provided by non-health professionals (e.g., community health worker, peer educator) or automatically delivered via website or mobile phone. Twelve studies delivered the interventions individually, 12 studies in a group format, and 21 studies employed a combination of individual and group formats. Twenty-six studies utilized technology in the delivery of interventions, through telephone, mobile phone, website, video, email, fax or surface mail. Ten studies had interventions offered in researcher-based locations (e.g., hospital, clinic, research institute) and 25 studies at participants’ home and surroundings (e.g., community setting, school, temple) or workplaces. The intervention duration ranged from 1.5 [26] to 72 [5] months. The total number of intervention sessions ranged from 1 [27] to 78 [28]. Most interventions (*n* = 34) were tailored through personalized goals or plans. Over half of the studies (*n* = 25) had a high level of intervention fidelity.

### 3.3. Risk of Bias Assessment

All studies had an overall high risk of bias, except two with some concerns [29,30] and three with low risk of bias [31,32,33]. The overall high risk of bias was mainly derived from the bias in deviations from intended interventions due to low adherence to the interventions (less than 80% of participants completing intervention sessions or intervention components). For the rest domains of the RoB 2 and ROBINS-I tools, most studies were rated as low risk of bias or some concerns, as described previously [8].

### 3.4. Intervention Effects

Meta-analyses showed lifestyle interventions resulted in significant improvement in diabetes incidence (RR 0.71, 95%CI [0.64, 0.79], *I*^2^ = 23.4%) and body weight (MD −2.13 kg, 95%CI [−2.71, −1.54], *I*^2^ = 86.7%) compared to control groups. Subgroup analyses by TIDieR components showed interventions with high number of sessions had a significantly greater reduction in diabetes incidence (RR 95%CI: 0.66 [0.57, 0.77] vs. 0.79 [0.69, 0.91], *P* = 0.043) and body weight (MD 95%CI: −2.79 kg [−3.58, −2.01] vs. −1.48 kg [−2.29, −0.66], *P* = 0.015) than those with low number of sessions (Table 1 and Table 2). None of the other TIDieR components were significantly associated with the outcomes (all *P* > 0.05).

Table 3 shows the association between intervention characteristics and the outcomes by ethnicity. For diabetes incidence, all studies that reported significant diabetes risk reduction had a high number of sessions (≥16 sessions) over a long duration (≥12 months), except two studies in the East and Southeast Asian group with a lower number of sessions (13 [33] and 14 sessions [34] respectively). For weight loss, most ethnic groups (i.e., European, South Asian, East and Southeast Asian, Middle Eastern) were more likely to report significant weight loss with interventions of short duration (<12 months). Some ethnic differences were seen in weight loss and number of sessions, in that 80% (8/10 studies) of the European group that reported significant weight loss had a high number of sessions, while 80% (4/5 studies) of the East and Southeast Asian group that reported significant weight loss had a low number of sessions.

Publication bias was found for body weight (Egger’s test *P* = 0.012) but not for diabetes incidence (Egger’s test *P* = 0.115). Funnel plots suggested smaller studies with greater weight loss were less likely to be published (Figure S2).

## 4. Discussion

This systematic review examined lifestyle intervention characteristics associated with T2DM prevention using the TIDieR framework in different ethnic groups, including in European, South Asian, East and Southeast Asian, Middle Eastern, Latin American and African groups. Meta-analysis showed lifestyle interventions with high number of sessions were significantly associated with a greater reduction in diabetes incidence and body weight. Other intervention characteristics such as intervention provider, delivery format and use of technology did not significantly alter diabetes incidence or body weight, suggesting these may be adapted according to contextual needs. Narrative synthesis showed a relatively high number of sessions over the long term is required to induce a significant diabetes risk reduction for all ethnic groups. Interventions of shorter duration appeared to be more effective in inducing weight loss in most ethnic groups, while the optimal number of sessions for weight loss might vary by ethnicity.

The associations of diabetes incidence and weight loss with number of sessions found in our meta-analyses are consistent with previous systematic reviews of real-world diabetes prevention programs, which showed each session attended was associated with 18% lower odds of developing T2DM and 26% or 0.15 kg more weight loss [13,16]. We additionally found across all ethnic groups, interventions that effectively reduced diabetes incidence were of a longer duration. T2DM is a progressive disease as a result of the complex interplay between insulin resistance and β-cell dysfunction [81]. The transition from early metabolic abnormalities (e.g., prediabetes) to T2DM may take many years [82]. Lifestyle interventions for T2DM prevention typically involve multiple health related goals (e.g., weight loss, increased physical activity, reduced total and saturated fat intake, increased fibre intake) with the number of goals achieved incrementally decreasing the risk of developing T2DM [83,84]. These may explain the reduction in diabetes incidence in interventions of longer follow-up period and higher number of sessions to facilitate the achievement of the multiple diabetes prevention lifestyle goals. Conversely, we found short-term interventions tended to be more effective for weight loss in most ethnic groups. This could be due to weight regain in longer-term studies, which may result from barriers to maintain healthy eating and physical activity behaviours over time [85]. It is documented that most participants in lifestyle programs will regain at least half the weight lost after 2 years and return to their baseline weight after 3 to 5 years [86]. Given weight loss is the primary driver of diabetes risk reduction [83], strategies should be developed to maintain long-term weight loss in each ethnic group. The only potential ethnic difference we noticed was a lower number of sessions associated with better weight loss outcomes in the East and Southeast Asian group. This may reflect a cultural preference, as greater adherence to didactically delivered lifestyle information was found in some Asian subgroups and thus may result in lower intervention dose requirement [87]. However, we were unable to determine the optimal number of sessions for different ethnic groups due to the limited number of studies in each ethnic group; this remains to be confirmed in future research.

This study has several limitations. First, ethnicity was inconsistently described across the studies and as such, we included additional proxies (e.g., race, country of birth, cultural background) to help define ethnicity. Second, the majority of included studies were rated as overall high risk of bias, mainly caused by suboptimal adherence to the interventions. Publication bias was also indicated for the weight outcome. Furthermore, due to the small number of studies in each category of intervention characteristics when stratified by ethnicity, the associations between intervention characteristics and the intervention effects in each ethnic group require further investigation.

In conclusion, this systematic review suggests a high number of sessions is associated with a greater reduction in diabetes incidence and body weight. There may be ethnic preferences for the optimal number of sessions. More research including engagement with stakeholders is needed to develop the most appropriate intervention strategies for T2DM prevention in different ethnic groups.

## Figures and Tables

**Table 1:** Subgroup analyses of lifestyle intervention on diabetes incidence by TIDieR components.

TIDieR Components	Studies ^a^	Risk Ratio (95%CI)	*I*^2^ (%)	*P* for Subgroup Differences
Use of theory				0.958
Yes	18	0.71 (0.59, 0.85)	32.5	
No	7	0.71 (0.61, 0.81)	6.7	
Intervention provider				0.334
With health professional	16	0.73 (0.65, 0.81)	5.6	
Without health professional	9	0.65 (0.48, 0.87)	56.0	
Intervention duration				0.538
Short (<12 months)	9	0.64 (0.40, 1.02)	0.0	
Long (≥12 months)	16	0.71 (0.62, 0.81)	40.3	
Number of sessions				0.043
Low (≤15 sessions)	12	0.79 (0.69, 0.91)	0.0	
High (≥16 sessions)	13	0.66 (0.57, 0.77)	31.9	
Delivery format				0.492
Group	5	0.71 (0.50, 1.00)	55.3	
Individual	11	0.77 (0.67, 0.88)	0.0	
Combined	9	0.66 (0.48, 0.90)	31.9	
Technology (e.g., phone, website)				0.177
With technology	14	0.77 (0.67, 0.90)	2.7	
In-person only	11	0.68 (0.57, 0.80)	32.0	
Location of intervention				0.899
Researcher-based	7	0.70 (0.60, 0.82)	7.9	
Participant-based	16	0.69 (0.58, 0.83)	33.3	
Combined	2	0.79 (0.03, 19.21)	42.6	
Tailoring				0.224
Yes	19	0.70 (0.60, 0.81)	40.6	
No	6	0.77 (0.71, 0.83)	0.0	
Fidelity				0.868
Low/medium	10	0.70 (0.64, 0.78)	0.0	
High	15	0.72 (0.57, 0.89)	45.5	

^a^ Number of studies by ethnic groups.

**Table 2:** Subgroup analyses of lifestyle intervention on body weight by TIDieR components.

TIDieR Components	Studies ^a^	Mean Difference, kg (95%CI)	*I*^2^ (%)	*P* for Subgroup Differences
Use of theory				0.280
Yes	34	−2.25 (−2.94, −1.55)	88.5	
No	8	−1.63 (−2.71, −0.55)	60.1	
Intervention provider				0.395
With health professional	30	−2.27 (−3.02, −1.51)	89.5	
Without health professional	12	−1.79 (−2.71, −0.87)	64.3	
Intervention duration				0.404
Short (<12 months)	25	−2.34 (−3.12, −1.55)	80.4	
Long (≥12 months)	17	−1.85 (−2.79, −0.90)	90.3	
Number of sessions				0.015
Low (≤15 sessions)	23	−1.48 (−2.29, −0.66)	85.0	
High (≥16 sessions)	19	−2.79 (−3.58, −2.01)	82.6	
Delivery format				0.996
Group	12	−2.15 (−3.55, −0.75)	91.9	
Individual	9	−2.09 (−3.19, −0.98)	69.5	
Combined	21	−2.13 (−3.01, −1.24)	84.6	
Technology (e.g., phone, website)				0.590
With technology	26	−2.25 (−2.98, −1.53)	83.6	
In-person only	16	−1.92 (−3.02, −0.81)	89.1	
Location of intervention				0.096
Researcher-based	8	−1.46 (−1.97, −0.94)	16.0	
Participant-based	24	−2.31 (−3.11, −1.51)	88.1	
Combined	10	−2.39 (−4.04, −0.74)	90.9	
Tailoring				0.911
Yes	31	−2.11 (−2.84, −1.38)	87.4	
No	11	−2.18 (−3.26, −1.09)	83.6	
Fidelity				0.271
Low/medium	16	−1.76 (−2.58, −0.94)	74.8	
High	26	−2.37 (−3.20, −1.54)	89.6	

^a^ Number of studies by ethnic groups.

**Table 3:** TIDieR components and the effects of lifestyle intervention on diabetes incidence and body weight by ethnicity.

Study	Ethnic Group	TIDieR Components									Effect	
		Theory Use	Intervention Provider	Intervention Duration	Number of Sessions	Delivery Format	Technology	Location	Tailoring	Fidelity	Diabetes Risk Reduction	Weight Loss
Aguiar et al. 2016 [27]; Rollo et al. 2017 [35]	European	Yes	No HP	Short	Low	Individual	Yes	P-based	Yes	High	NR	Sig
Block et al. 2015 [22]; Block et al. 2016 [36]	European	Yes	No HP	Short	High	Individual	Yes	P-based	Yes	High	Not sig	Sig
Cheung et al. 2019 [23]	European	Yes	HP	Short	High	Individual	Yes	P-based	Yes	High	NR	NR
Davies et al. 2016 [37]	European	Yes	HP	Long	Low	Combined	Yes	Combined	Yes	High	Not sig	Not sig
Duijzer et al. 2017 [31]	European	Yes	HP	Long	High	Combined	Yes	Combined	Yes	High	NR	Sig
Heideman et al. 2015 [32]	European	Yes	HP	Short	Low	Combined	Yes	Combined	Yes	High	NR	Not sig
Holmes et al. 2018 [38]	European	Yes	HP	Short	High	Combined	Yes	Combined	Yes	High	NR	Sig
Juul et al. 2016 [39]	European	Yes	HP	Short	Low	Group	No	R-based	Yes	Medium	NR	Not sig
Knowler et al. 2002 [4]; West et al. 2008 [30]	European	Yes	HP	Long	High	Combined	Yes	Combined	Yes	High	Sig	Sig
Kramer et al. 2015 [40]	European	Yes	HP	Short	High	Combined	Yes	P-based	Yes	High	NR	Sig
Kramer et al. 2018 [41]	European	Yes	HP	Short	High	Combined	Yes	P-based	Yes	High	NR	Sig
O’Reilly et al. 2016 [24]; O’Reilly et al. 2019 [42]	European	Yes	HP	Short	Low	Combined	Yes	P-based	Yes	High	Not sig	Not sig
Peacock et al. 2015 [43]	European	Yes	HP	Short	Low	Combined	Yes	Combined	Yes	High	NR	Sig
Roumen et al. 2008 [44]; Roumen et al. 2011 [45]; den Boer et al. 2013 [46]	European	No	HP	Long	High	Combined	No	R-based	Yes	Medium	Sig	Sig
Weinhold et al. 2015 [47]; Miller et al. 2015 [48]; Miller et al. 2016 [49]	European	Yes	HP	Short	High	Group	No	P-based	Yes	High	NR	Sig
Yates et al. 2017 [50]	European	Yes	HP	Long	Low	Combined	Yes	Combined	Yes	High	NR	Not sig
Block et al. 2015 [22]; Block et al. 2016 [36]	South Asian	Yes	No HP	Short	High	Individual	Yes	P-based	Yes	High	Not sig	Not sig
Cheung et al. 2019 [23]	South Asian	Yes	HP	Short	High	Individual	Yes	P-based	Yes	High	NR	NR
Fottrell et al. 2019 [51]	South Asian	Yes	No HP	Long	High	Group	No	P-based	Yes	High	Sig	Not sig
Islam et al. 2014 [52]; Lim et al. 2019 [53]	South Asian	Yes	No HP	Short	Low	Combined	Yes	P-based	Yes	Medium	NR	Sig
Limaye et al. 2017 [54]	South Asian	No	No HP	Long	High	Individual	Yes	P-based	No	High	NR	Sig
Muralidharan et al. 2019 [55]	South Asian	Yes	HP	Short	Low	Individual	Yes	P-based	No	High	Not sig	Sig
Nanditha et al. 2020 [56]	South Asian	Yes	No HP	Long	High	Individual	Yes	P-based	Yes	High	Not sig	NR
Patel et al. 2017 [57]	South Asian	Yes	HP	Short	Low	Group	Yes	P-based	Yes	Medium	NR	Not sig
Ramachandran et al. 2006 [6]; Snehalatha et al. 2008 [58]	South Asian	No	HP	Long	High	Individual	Yes	P-based	Yes	Low	Sig	Not sig
Ramachandran et al. 2013 [28]; Ram et al. 2014 [59]; Nanditha et al. 2018 [60]	South Asian	Yes	No HP	Long	High	Individual	Yes	P-based	Yes	High	Sig	NR
Thankappan et al. 2018 [61]; Lotfaliany et al. 2020 [62]	South Asian	Yes	HP	Long	Low	Group	No	P-based	Yes	High	Not sig	Not sig
Weber et al. 2016 [63]	South Asian	Yes	HP	Short	High	Group	No	R-based	No	Medium	Not sig	Sig
Aekplakorn et al. 2019 [34]	East and Southeast Asian	No	HP	Long	Low	Group	No	R-based	No	Medium	Sig	Sig
Bender et al. 2018 [64]	East and Southeast Asian	Yes	HP	Short	Low	Combined	Yes	Combined	Yes	Low	NR	Sig
Block et al. 2015 [22]; Block et al. 2016 [36]	East and Southeast Asian	Yes	No HP	Short	High	Individual	Yes	P-based	Yes	High	Not sig	Sig
Ibrahim et al. 2016 [33]	East and Southeast Asian	Yes	HP	Long	Low	Combined	Yes	P-based	Yes	High	Sig	Sig
Inouye et al. 2014 [29]	East and Southeast Asian	Yes	HP	Short	Low	Group	No	P-based	Yes	High	NR	Sig
Islam et al. 2013 [65]	East and Southeast Asian	Yes	No HP	Short	Low	Combined	Yes	P-based	Yes	Medium	NR	Not sig
Moungngern et al. 2018 [66]	East and Southeast Asian	Yes	HP	Short	Low	Combined	Yes	Combined	No	Low	NR	Not sig
Pan et al. 1995 [67]; Pan et al. 1997 [5]; Li et al. 2008 [68]	East and Southeast Asian	No	HP	Long	High	Combined	No	R-based	Yes	Medium	Sig	Not sig
Sakane et al. 2011 [69]; Sakane et al. 2014 [70]	East and Southeast Asian	Yes	HP	Long	Low	Combined	Yes	Combined	Yes	Medium	Not sig	Not sig
Shek et al. 2014 [71]	East and Southeast Asian	No	HP	Long	Low	Individual	No	R-based	Yes	Low	Not sig	NR
Wong et al. 2013 [72]	East and Southeast Asian	Yes	No HP	Long	High	Individual	Yes	P-based	No	High	Not sig	Not sig
Abujudeh et al. 2012 [73]	Middle Eastern	No	HP	Short	High	Group	No	P-based	No	Low	NR	Sig
Al-Hamdan et al. 2019 [74]	Middle Eastern	No	HP	Short	Low	Individual	No	R-based	Yes	Low	NR	NR
Amer et al. 2020 [75]	Middle Eastern	No	HP	Long	Low	Individual	No	R-based	Yes	Low	Not sig	Not sig
Zilberman-Kravits et al. 2018 [76]	Middle Eastern	No	HP	Long	Low	Combined	No	R-based	No	Low	Not sig	Not sig
Block et al. 2015 [22]; Block et al. 2016 [36]	Latin American	Yes	No HP	Short	High	Individual	Yes	P-based	Yes	High	Not sig	Not sig
Knowler et al. 2002 [4]; West et al. 2008 [30]	Latin American	Yes	HP	Long	High	Combined	Yes	Combined	Yes	High	Sig	Sig
Ockene et al. 2012 [77]	Latin American	Yes	No HP	Long	High	Combined	No	P-based	Yes	High	Not sig	Sig
Parikh et al. 2010 [78]	Latin American	Yes	No HP	Short	Low	Group	No	P-based	No	Medium	Not sig	Sig
Van Name et al. 2016 [79]	Latin American	Yes	HP	Short	Low	Group	No	P-based	No	Medium	Not sig	Sig
Auslander et al. 2000 [80]; Auslander et al. 2002 [25]	African	Yes	No HP	Short	Low	Combined	No	P-based	Yes	High	NR	Not sig
Bernstein et al. 2014 [26]	African	No	HP	Short	Low	Group	No	R-based	No	Medium	NR	Not sig
Knowler et al. 2002 [4]; West et al. 2008 [30]	African	Yes	HP	Long	High	Combined	Yes	Combined	Yes	High	Sig	Sig
O’Reilly et al. 2016 [24]; O’Reilly et al. 2019 [42]	African	Yes	HP	Short	Low	Combined	Yes	P-based	Yes	High	Not sig	Not sig

HP, health professional; P, participant; R, researcher; NR, not reported; Sig, significant reduction in the intervention group compared to the control group (*P* < 0.05).

## Data Availability

The data presented in this study are available in the article and Supplementary Materials.

## Supplementary Materials

The following are available online at https://www.mdpi.com/article/10.3390/nu13114118/s1, Table S1: coding of the TIDieR components, Table S2: intervention characteristics of included studies according to the TIDieR checklist, Figure S1: flow diagram of included studies, Figure S2: funnel plots for publication bias.
