# Supplementary material for: The Effect of Lifestyle Intervention on Diabetes Prevention by Ethnicity: A Systematic Review of Intervention Characteristics Using the TIDieR Framework
Source: Nutrients. 2021 Nov 17;13(11):4118. doi: 10.3390/nu13114118 (PMC8624360; doi:10.3390/nu13114118)
Supplement: Supplementary file 1 [file nutrients-13-04118-s001.zip › nutrients-1413766-supplementary.pdf]

Title: The effect of lifestyle intervention on diabetes prevention by ethnicity: a systematic review of intervention characteristics using the TIDieR framework

Supplementary material:

Table S1. Coding of the TIDieR components

Table S2. Intervention characteristics of included studies according to the TIDieR checklist

Figure S1. Flow diagram of included studies

Figure S2. Funnel plots for publication bias

References for Table S1-2

Table S1. Coding of the TIDieR components

TIDieR components	Description	Coding details
Use of theory	Use of any theory that underpins the intervention	Yes; no
Intervention type	What information delivered in the intervention	Diet; physical activity; combined
Intervention provider	Who involved in providing the intervention	Health professional = health professional involved (e.g. nurse, dietitian, physician); no health professional = no health professional involved (e.g. peer educator, automated delivery via mobile phone)
Intervention duration	The duration of the intervention	Short = less than 12 months; long = 12 months or more
Number of sessions	The total number of intervention sessions was calculated based on different types of contacts [1]:<br>1 in-person group/individual session = 1 session;<br>1 online/telephone session = 0.5 session;<br>1 text/email/fax/newsletter contact = 0.25 session	Low = 15 sessions or less; high = 16 sessions or more
Delivery format	Whether the intervention delivered individually or in a group	Group; individual; combined
Technology	Use of technology to deliver the intervention by distance, such as by telephone, surface mail, email, internet, DVD, etc.	Yes = with technology; no = in-person only
Location of intervention	Where the intervention occurred	Researcher-based (e.g. research centre, clinic, hospital); participant-based (e.g. home, worksite, community); combined
Tailoring	Whether the intervention planned to be personalised or titrated so that not all participants received an identical intervention	Yes; no
Fidelity	The use of standard curriculum for the delivery of intervention and quality assurance measures (e.g. checklist) to monitor the implementation of the intervention [2]	Low = no standard curriculum; medium = a standard curriculum followed but no quality assurance measures; high = a standard curriculum followed and quality assurance measures reported

Table S2. Intervention characteristics of included studies according to the TIDieR checklist

Author, year	Ethnic group	Theory; Tailoring	Intervention type; Intervention provider	Delivery format; Location of intervention	Intervention duration; Number of sessions	Technology used; Fidelity
Abujudeh et al 2012 [3]	Middle Eastern	No theory reported;<br><br>No tailoring reported	Diet and physical activity;<br><br>Nutritionist and physiotherapist	Group;<br><br>Worksite	6 months;<br><br>In-person: 17	No technology used;<br><br>Low fidelity
Aekplakorn et al 2019 [4]	East and Southeast Asian	No theory reported;<br><br>No tailoring reported	Diet and physical activity;<br><br>Health care provider	Group;<br><br>Primary care practice	24 months;<br><br>In-person: 14	No technology used;<br><br>Medium fidelity
Aguiar et al 2016;<br>Rollo et al 2017 [5, 6]	European	Bandura's social cognitive theory;<br><br>Personal goal setting	Diet and physical activity;<br><br>Participant self-administered after an initial individual 15-min orientation to the intervention resources	Individual;<br><br>Home	6 months;<br><br>In-person: 1	DVD and website;<br><br>High fidelity
Al-Hamdan et al 2019 [7]	Middle Eastern	No theory reported;<br><br>Individualised counselling	Diet and physical activity;<br><br>Nutritionist	Individual;<br><br>Primary health clinic	3 months;<br><br>In-person: 6	No technology used;<br><br>Low fidelity
Amer et al 2020 [8]	Middle Eastern	No theory reported;<br><br>Personalised lifestyle counselling	Diet and physical activity;<br><br>Nutritionist	Individual;<br><br>Hospital	18 months;<br><br>In-person: 6	No technology used;<br><br>Low fidelity

Auslander et al 2000;<br>Auslander et al 2002 [9, 10]	African	The stages of change theory;<br><br>Tailored to the participants’ stage of change for individual dietary patterns	Diet;<br><br>Peer educator	Combined;<br><br>Community	3 months;<br><br>In-person: 12	No technology used;<br><br>High fidelity
Bender et al 2018 [11]	East and Southeast Asian	Social cognitive learning theory;<br><br>Individual tailored goals for weight, diet and physical activity	Diet and physical activity;<br><br>Research staff	Combined;<br><br>Office and home	3 months;<br><br>In-person: 4<br>Facebook post: 12	Mobile phone;<br><br>Low fidelity
Bernstein et al 2014 [12]	African	No theory reported;<br><br>No tailoring reported	Diet and physical activity;<br><br>Dietitian, chef and behavioural health specialist	Group;<br><br>Family health centre	6 weeks;<br><br>In-person: 6	No technology used;<br><br>Medium fidelity
Block et al 2015;<br>Block et al 2016 [13, 14]	European, East and Southeast Asian, Latin American, South Asian	Models centering on cues and triggers, social cognitive theory, the theory of planned behaviour, behavioural economics, and positive psychology;<br><br>Individual tailored goal setting and tailored	Diet and physical activity;<br><br>Delivered via the Web, email, mobile phone and automated phone calls	Individual;<br><br>Home	6 months;<br><br>Email: 48<br>Phone: 12	Website, email and mobile phone;<br><br>High fidelity

		behavioural support				
Cheung et al 2019 [15]	South Asian, European	A range of theoretical frameworks, such as information-motivation-behavioural skills model, the theory of reasoned action, the theory of planned behaviour, social cognitive theory, control theory, and operant conditioning;<br><br>Semi-personalised messages customized for women who were not breastfeeding, or were South Asian	Diet and physical activity;<br><br>Digital health support program complemented by one diet counselling session by dietitian	Individual;<br><br>Home	6 months;<br><br>SMS: 104<br>Phone: 1	Mobile phone;<br><br>High fidelity
Davies et al 2016 [16]	European	A theoretical basis with a philosophy centred on patient empowerment;<br><br>Personalised step-per-day goals	Diet and physical activity;<br><br>Health educator	Combined;<br><br>Primary care practice and home	36 months;<br><br>In-person: 3<br>Phone: 12	Telephone;<br><br>High fidelity
Duijzer et al 2017 [17]	European	A combination of theories, such as Stages of Change model and Theory of Planned Behaviour;	Diet and physical activity;<br><br>Primary healthcare professional (general practitioner, practice	Combined;<br><br>Primary care practice, home and sports clinic	12 months;<br><br>In-person: 48.9 on average<br>Phone: 2	Telephone;<br><br>High fidelity

		Tailored to participants' individual needs	nurse, dietitian and physiotherapist)			
Fottrell et al 2019 [18]	South Asian	Participatory learning and action approach;<br><br>Strategies to address communities' own barriers	Diet and physical activity;<br><br>Lay facilitator	Group;<br><br>Community	18 months;<br><br>In-person: 18	No technology used;<br><br>High fidelity
Heideman et al 2015 [19]	European	Health Action Process Approach;<br><br>Personal action plan	Diet and physical activity;<br><br>Dietitian	Combined;<br><br>Primary care practice or outpatient clinic, and home	30 weeks;<br><br>In-person: 2<br>Newsletter: 4	Surface mail;<br><br>High fidelity
Holmes et al 2018 [20]	European	The person-centred philosophy;<br><br>Each session adapted to the needs of the group based on socioeconomic status and ethnicity, and individual weight loss targets	Diet and physical activity;<br><br>Health educator and commercial weight management organization	Combined;<br><br>Hospital, commercial weight management organization, and home	6 months;<br><br>In-person: 13<br>Phone: 6<br>SMS: 14	Telephone and text messaging;<br><br>High fidelity
Ibrahim et al 2016 [21]	East and Southeast Asian	Health Belief Model;<br><br>Individualised diet plan, weight loss goals and	Diet and physical activity;<br><br>Dietitian, researcher and community volunteer	Combined;<br><br>Community (community hall or	12 months;<br><br>In-person: 12<br>Phone: 2	Telephone;<br><br>High fidelity

		behaviour modification		recreational park) and home		
Inouye et al 2014 [22]	East and Southeast Asian	Social learning theory, community-based participatory research approach;<br><br>Personal behavioural goals	Diet and physical activity;<br><br>Health care worker	Group;<br><br>Community (in a community park)	6 months;<br><br>In-person: 8	No technology used;<br><br>High fidelity
Islam et al 2013 [23]	East and Southeast Asian	Community-based participatory research approach;<br><br>Individualised strategies and goal-setting	Diet and physical activity;<br><br>Community health worker	Combined;<br><br>Community (in a convenient community setting) and home	6 months;<br><br>In-person: 6<br>Phone: 10	Telephone;<br><br>Medium fidelity
Islam et al 2014;<br>Lim et al 2019 [24, 25]	South Asian	Community-based participatory research approach;<br><br>Individualised strategies and goal-setting	Diet and physical activity;<br><br>Community health worker	Combined;<br><br>Community (in a convenient community setting) and home	6 months;<br><br>In-person: 6<br>Phone: 10	Telephone;<br><br>Medium fidelity
Juul et al 2016 [26]	European	Mezirow's theory of transformative learning, Health Literacy Theory and dimensions of health knowledge and action competence;	Diet and physical activity;<br><br>Dietitian and occupational therapist	Group;<br><br>Health care centre	6 months;<br><br>In-person: 6	No technology used;<br><br>Medium fidelity

		Personal goal setting				
Knowler et al 2002;<br>West et al 2008 [27, 28]	European, African, Latin American	Social cognitive theory;<br><br>Individualised counselling	Diet and physical activity;<br><br>Case manager (called "lifestyle coach", the majority were registered dietitians)	Combined;<br><br>Clinic and home	2.8 years on average;<br><br>In-person: 29.8 on average<br>Phone: 13.8 on average	Phone;<br><br>High fidelity
Kramer et al 2015 [29]	European	Social cognitive theory;<br><br>Personal behavioural goals	Diet and physical activity;<br><br>Lifestyle coach	Combined;<br><br>Worksite and home	6 months;<br><br>In-person: 16 (16 core sessions delivered either face-to-face or via DVD with phone call support)	DVD and telephone;<br><br>High fidelity
Kramer et al 2018 [30]	European	Social cognitive theory;<br><br>Personal behavioural goals	Diet and physical activity;<br><br>Lifestyle coach (i.e. dietitian and exercise specialist)	Combined;<br><br>Community centre	6 months;<br><br>In-person: 16 (16 core sessions delivered either face-to-face or via DVD with phone call support)	DVD and telephone;<br><br>High fidelity
Limaye et al 2017 [31]	South Asian	No theory reported;<br><br>No tailoring reported	Diet and physical activity;<br><br>Delivered via mobile phone messages and	Individual;<br><br>Worksite and home	1 year;<br><br>SMS: 150<br>Email: 100	Mobile phone and email;<br><br>High fidelity

			emails			
Moungngern et al 2018 [32]	East and Southeast Asian	Health Belief Model, Self-Efficacy Theory, and group process techniques;<br><br>No tailoring reported	Diet and physical activity;<br><br>Nurse	Combined;<br><br>Hospital and home	6 months;<br><br>In-person: 3<br>Phone: 8	Telephone;<br><br>Low fidelity
Muralidharan et al 2019 [33]	South Asian	Social cognitive theory;<br><br>No tailoring reported	Diet and physical activity;<br><br>Delivered via a mobile phone app along with weekly coach calls by nutritionist	Individual;<br><br>Home	12 weeks;<br><br>Video lesson: 12<br>Phone: 12<br>Text message: according to the progress of each user	Mobile phone;<br><br>High fidelity
Nanditha et al 2020 [34]	South Asian	The transtheoretical model of behavioural change;<br><br>Individual tailored SMS messages	Diet and physical activity;<br><br>Delivered via SMS messages	Individual;<br><br>Worksite and home	2 years;<br><br>SMS: 208-312	Mobile phone;<br><br>High fidelity
Ockene et al 2012 [35]	Latin American	Social cognitive theory and patient-centred counselling;<br><br>Personal goal setting and solutions	Diet and physical activity;<br><br>Community individual	Combined;<br><br>The senior centre and home	12 months;<br><br>In-person: 16	No technology used;<br><br>High fidelity
O'Reilly et al 2016;	European,	Health Action Process	Diet and physical activity;	Combined;	9 months;	Telephone;

O'Reilly et al 2019 [36, 37]	African	Approach, social cognitive and self-regulation theory;<br><br>Individual goal setting	Healthcare professional	Home and community venue (community settings close to the woman's home)	In-person: 6<br>Phone: 2	High fidelity
Pan et al 1995;<br>Pan et al 1997;<br>Li et al 2008 [38-40]	East and Southeast Asian	No theory reported;<br><br>Personal goal setting, and individualised counselling based on BMI, age, past exercise patterns, and health problems	Diet and physical activity;<br><br>Physician	Combined;<br><br>Primary care clinic	6 years;<br><br>In-person: 29	No technology used;<br><br>Medium fidelity
Parikh et al 2010 [41]	Latin American	Self-efficacy theory, community-based participatory research approach;<br><br>No tailoring reported	Diet and physical activity;<br><br>Peer educator	Group;<br><br>Community (community sites, often where recruitment occurred)	10 weeks;<br><br>In-person: 8	No technology used;<br><br>Medium fidelity
Patel et al 2017 [42]	South Asian	Community-based participatory research framework;<br><br>Personal SMART goals	Diet and physical activity;<br><br>Healthcare professional	Group;<br><br>Hindu temple or mandir	12 weeks;<br><br>In-person: 12	Text messaging app and email;<br><br>Medium fidelity
Peacock et al 2015 [43]	European	Behaviour change theory;	Diet and physical activity;	Combined;	3 months;	Website;

		Weekly goals based on the previous weeks steps	Web-based program and a four-week nutrition coaching workshop by dietitian	Hospital and home	In-person: 4<br>Website: 12	High fidelity
Ramachandran et al 2006;<br>Snehalatha et al 2008 [44, 45]	South Asian	No theory reported;<br><br>Individual advice based on occupation, mode of transport to work and leisure activities	Diet and physical activity;<br><br>Physician, dietitian and social worker	Individual;<br><br>Worksite and home	3 years;<br><br>In-person: 7<br>Phone: 36	Telephone;<br><br>Low fidelity
Ramachandran et al 2013;<br>Ram et al 2014;<br>Nanditha et al 2018 [46-48]	South Asian	The transtheoretical model of behavioural change;<br><br>Individual tailored mobile phone messaging	Diet and physical activity;<br><br>Delivered via mobile phone messages	Individual;<br><br>Worksite and home	2 years;<br><br>SMS: 312	Mobile phone;<br><br>High fidelity
Roumen et al 2008;<br>Roumen et al 2011;<br>den Boer et al 2013 [49-51]	European	No theory reported;<br><br>Personal goal setting and individualised advice	Diet and physical activity;<br><br>Dietitian and exercise trainer	Combined;<br><br>University	3 years;<br><br>In-person: 21	No technology used;<br><br>Medium fidelity
Sakane et al 2011;<br>Sakane et al 2014 [52, 53]	East and Southeast Asian	Theoretical concepts and techniques for behavioural change, such as self-efficacy, self-monitoring, and the transtheoretical model;<br><br>Personalised goals	Diet and physical activity;<br><br>Nurse	Combined;<br><br>Primary care centre and home	3 years;<br><br>In-person: 6<br>Phone: 4<br>Fax: 12	Telephone and fax;<br><br>Medium fidelity

Shek et al 2014 [54]	East and Southeast Asian	No theory reported;<br><br>Dietary advice based on participants' ideal body weight	Diet and physical activity;<br><br>Dietitian and nurse	Individual;<br><br>Hospital	36 months;<br><br>In-person: 8	No technology used;<br><br>Low fidelity
Thankappan et al 2018;<br>Lotfaliany et al 2020 [55, 56]	South Asian	Health Action Process Approach model;<br><br>Personal SMART goals	Diet and physical activity;<br><br>K-DPP team, expert panel and peer leader	Group;<br><br>Community (in a convenient neighborhood facility, e.g. schools, community halls)	12 months;<br><br>In-person: 15	No technology used;<br><br>High fidelity
Van Name et al 2016 [57]	Latin American	Social cognitive theory;<br><br>No tailoring reported	Diet and physical activity;<br><br>Nurse	Group;<br><br>School (in the classrooms and cafeteria of a public school near the health centre)	14 weeks;<br><br>In-person: 14	No technology used;<br><br>Medium fidelity
Weber et al 2016 [58]	South Asian	Social cognitive theory;<br><br>No tailoring reported	Diet and physical activity;<br><br>Health coach, fitness instructor and community volunteer peer leader	Group;<br><br>Diabetes care and research institution	4 months;<br><br>In-person: 16	No technology used;<br><br>Medium fidelity
Weinhold et al 2015;<br>Miller et al 2015;<br>Miller et al 2016 [59-61]	European	Social cognitive theory;<br><br>Individual behavioural goals and individualised	Diet and physical activity;<br><br>Dietitian	Group;<br><br>Worksite	16 weeks;<br><br>In-person: 16	No technology used;<br><br>High fidelity

		feedback				
Wong et al 2013 [62]	East and Southeast Asian	The theory of planned behaviour and the social cognitive theory behaviour;<br><br>No tailoring reported	Diet and physical activity;<br><br>Delivered via SMS messages	Individual;<br><br>Worksite and home	24 months;<br><br>SMS: 66	Mobile phone;<br><br>High fidelity
Yates et al 2017 [63]	European	An integrated theoretical framework, including Bandura's social cognitive theory, Gollwitzer's implementation intentions, Leventhal's common sense model, Chaiken's dual process theory, the person-centred philosophy and learning techniques;<br><br>Personal action plan	Diet and physical activity;<br><br>Healthcare professional	Combined;<br><br>General practice, local hospital, community settings (e.g. church halls), and home	30 months;<br><br>In-person: 3<br>Phone: 3	Telephone;<br><br>High fidelity
Zilberman-Kravits et al 2018 [64]	Middle Eastern	No theory reported;<br><br>No tailoring reported	Diet and physical activity;<br><br>Nurse, dietitian and sports instructor	Combined;<br><br>Clinic	24 months;<br><br>In-person: 7	No technology used;<br><br>Low fidelity

Figure S1. Flow diagram of included studies

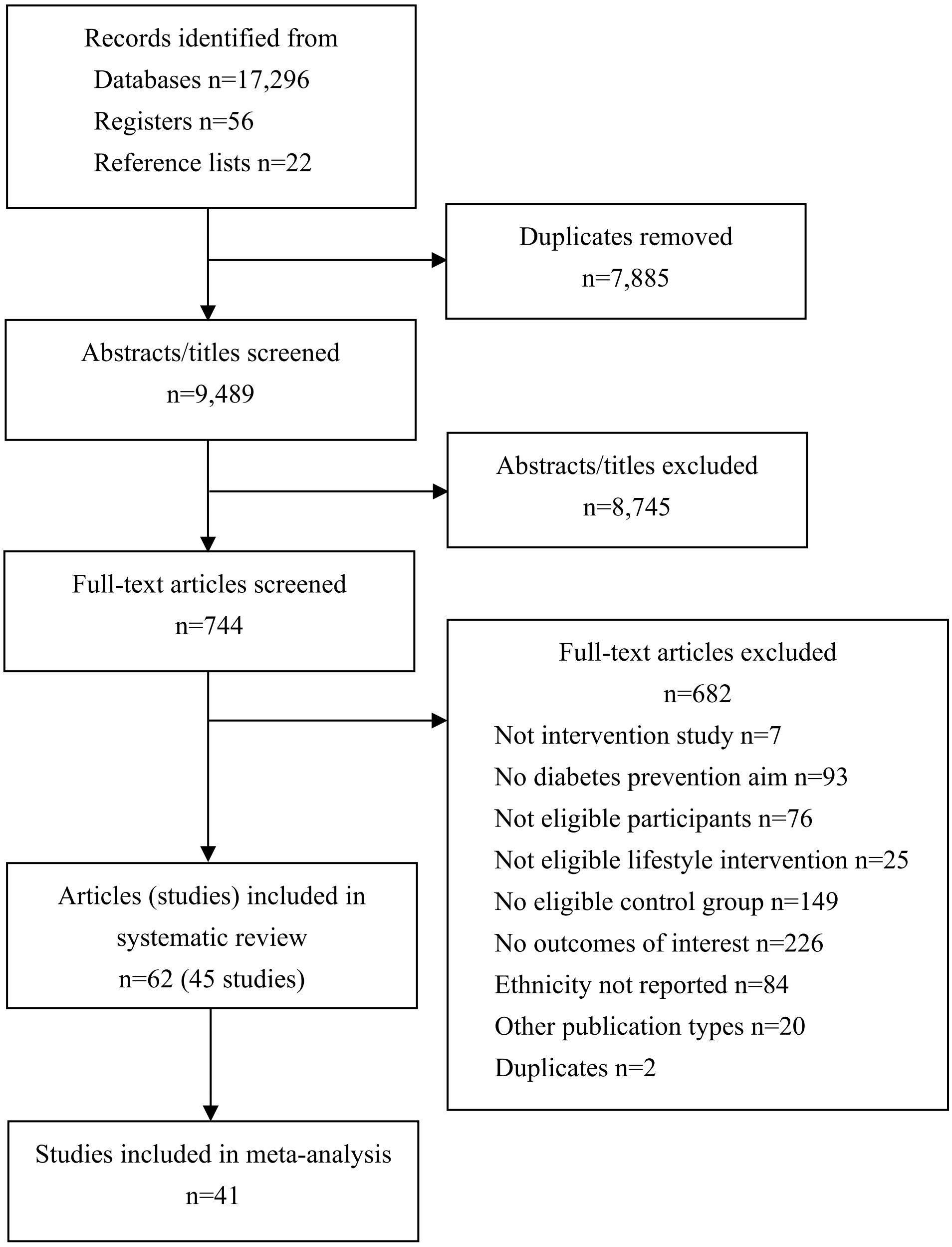

Figure S2. Funnel plots for publication bias among studies reporting diabetes incidence (A) and body weight (B)

A. Diabetes incidence

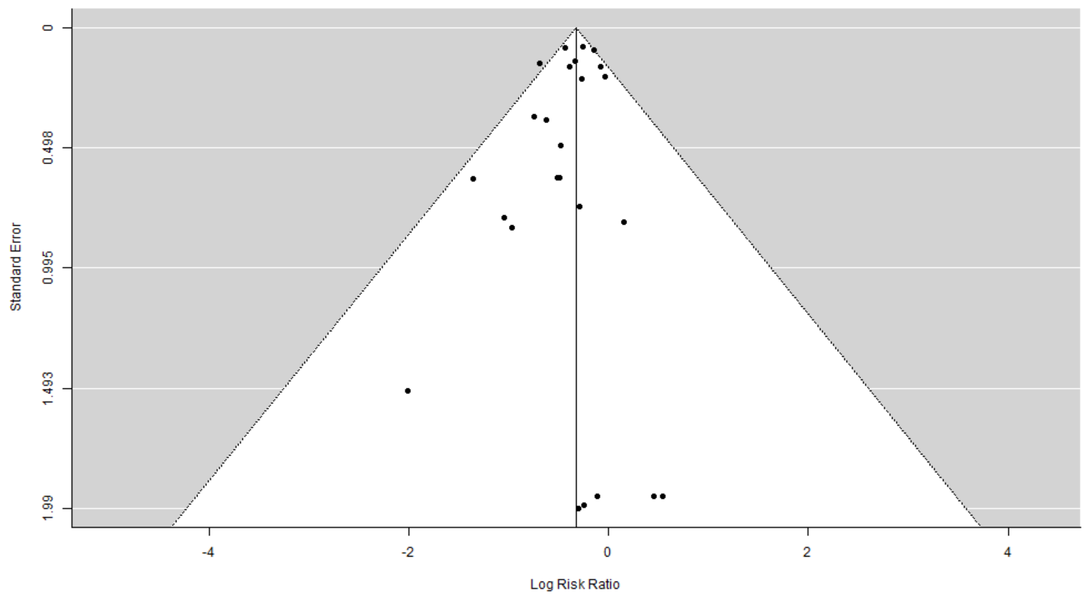

B. Body weight (kg)

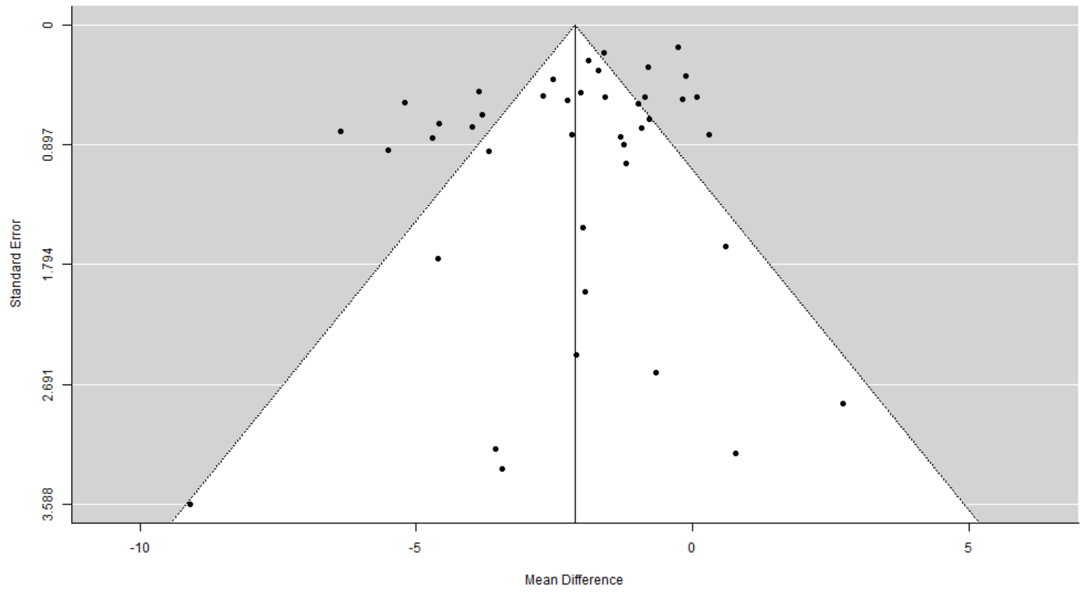

Egger's tests: *P*=0.115 (A) and *P*=0.012 (B)

References

[1] Lim S, Liang X, Hill B, Teede H, Moran LJ, O'Reilly S. A systematic review and meta-analysis of intervention characteristics in postpartum weight management using the TIDieR framework: A summary of evidence to inform implementation. Obes Rev. 2019;20:1045-56.

[2] Aziz Z, Absetz P, Oldroyd J, Pronk NP, Oldenburg B. A systematic review of real-world diabetes prevention programs: learnings from the last 15 years. Implement Sci. 2015;10:172.

[3] Abujudeh BA, Abu Al Rub RF, Al-Faouri IG, Gharaibeh MK. The impact of lifestyle modification in preventing or delaying the progression of type 2 diabetes mellitus among high-risk people in Jordan. Journal of Research in Nursing. 2012;17:32-44.

[4] Aekplakorn W, Tantayotai V, Numsangkul S, Tatsato N, Luckanajantachote P, Himathongkam T. Evaluation of a Community-Based Diabetes Prevention Program in Thailand: a Cluster Randomized Controlled Trial. Journal of primary care & community health. 2019;10:1-8.

[5] Aguiar EJ, Morgan PJ, Collins CE, Plotnikoff RC, Young MD, Callister R. Efficacy of the type 2 diabetes prevention using lifestyle education program RCT. American Journal of Preventive Medicine. 2016;50:353-64.

[6] Rollo ME, Aguiar EJ, Pursey KM, Morgan PJ, Plotnikoff RC, Young MD, et al. Impact on dietary intake of a self-directed, gender-tailored diabetes prevention program in men. World journal of diabetes. 2017;8:414-21.

[7] Al-Hamdan R, Avery A, Salter A, Al-Disi D, Al-Daghri NM, McCullough F. Identification of Education Models to Improve Health Outcomes in Arab Women with Pre-Diabetes. Nutrients. 2019;11:1113.

[8] Amer OE, Sabico S, Alfawaz HA, Aljohani N, Hussain SD, Alnaami AM, et al. Reversal of Prediabetes in Saudi Adults: Results from an 18 Month Lifestyle Intervention. Nutrients. 2020;12:18.

[9] Auslander W, Haire-Joshu D, Houston C, Rhee CW, Williams JH. A controlled evaluation of staging dietary patterns to reduce the risk of diabetes in African-American women. Diabetes Care. 2002;25:809-14.

[10] Auslander W, Haire-Joshu D, Houston C, Williams JH, Krebill H. The short-term impact of a health promotion program for low-income African American women. Research on Social Work Practice. 2000;10:78-97.

[11] Bender MS, Cooper BA, Flowers E, Ma R, Arai S. Filipinos Fit and Trim - A feasible and efficacious DPP-based intervention trial. Contemporary clinical trials communications. 2018;12:76-84.

[12] Bernstein A, Gendy G, Rudd N, Doyle J, Fay S, Moffett K, et al. Management of prediabetes through lifestyle modification in overweight and obese African-American women: The Fitness, Relaxation, and Eating to Stay Healthy (FRESH) randomized controlled trial. Public Health. 2014;128:674-7.

[13] Block G, Azar KM, Romanelli RJ, Block TJ, Hopkins D, Carpenter HA, et al. Diabetes prevention and weight loss with a fully automated behavioral intervention by email, Web, and mobile phone: A randomized controlled trial among persons with prediabetes. Journal of Medical Internet Research. 2015;17:e240.

[14] Block G, Azar KM, Romanelli RJ, Block TJ, Palaniappan LP, Dolginsky M, et al. Improving diet, activity and wellness in adults at risk of diabetes: randomized controlled trial. Nutrition & diabetes. 2016;6:e231.

[15] Cheung NW, Blumenthal C, Smith BJ, Hogan R, Thiagalingam A, Redfern J, et al. A Pilot Randomised Controlled Trial of a Text Messaging Intervention with Customisation Using Linked Data from Wireless Wearable Activity Monitors to Improve Risk Factors Following Gestational Diabetes. Nutrients. 2019;11:590.

[16] Davies MJ, Gray LJ, Troughton J, Gray A, Tuomilehto J, Farooqi A, et al. A community based primary prevention programme for type 2 diabetes integrating identification and lifestyle intervention for prevention: The Let's Prevent Diabetes cluster randomised controlled trial. Preventive Medicine: An International Journal Devoted to Practice and Theory. 2016;84:48-56.

[17] Duijzer G, Haveman-Nies A, Jansen SC, Beek JT, van Bruggen R, Willink MGJ, et al. Effect and maintenance of the SLIMMER diabetes prevention lifestyle intervention in Dutch primary healthcare: a randomised controlled

trial. Nutrition & diabetes. 2017;7:e268.
[18] Fottrell E, Ahmed N, Morrison J, Kuddus A, Shaha SK, King C, et al. Community groups or mobile phone messaging to prevent and control type 2 diabetes and intermediate hyperglycaemia in Bangladesh (DMagic): a cluster-randomised controlled trial. The lancet diabetes and endocrinology. 2019;7:200-12.
[19] Heideman WH, de Wit M, Middelkoop BJ, Nierkens V, Stronks K, Verhoeff AP, et al. Diabetes risk reduction in overweight first degree relatives of type 2 diabetes patients: Effects of a low-intensive lifestyle education program (DiAlert): A randomized controlled trial. Patient Education and Counseling. 2015;98:476-83.
[20] Holmes VA, Draffin CR, Patterson CC, Francis L, Irwin J, McConnell M, et al. Postnatal Lifestyle Intervention for Overweight Women With Previous Gestational Diabetes: a Randomized Controlled Trial. Journal of clinical endocrinology and metabolism. 2018;103:2478-87.
[21] Ibrahim N, Moy FM, Awalludin IAN, Ali ZM, Ismail IS. Effects of a community-based healthy lifestyle intervention program (Co-HELP) among adults with prediabetes in a developing country: a quasi-experimental study. Plos one. 2016;11:e0167123.
[22] Inouye J, Matsuura C, Li D, Castro R, Leake A. Lifestyle intervention for Filipino Americans at risk for diabetes. Journal of Community Health Nursing. 2014;31:225-37.
[23] Islam NS, Zanowiak JM, Wyatt LC, Chun K, Lee L, Kwon SC, et al. A randomized-controlled, pilot intervention on diabetes prevention and healthy lifestyles in the New York City Korean community. J Community Health. 2013;38:1030-41.
[24] Islam NS, Zanowiak JM, Wyatt LC, Kavathe R, Singh H, Kwon SC, et al. Diabetes prevention in the New York City Sikh Asian Indian community: a pilot study. Int J Environ Res Public Health. 2014;11:5462-86.
[25] Lim S, Wyatt LC, Chauhan H, Zanowiak JM, Kavathe R, Singh H, et al. A Culturally Adapted Diabetes Prevention Intervention in the New York City Sikh Asian Indian Community Leads to Improvements in Health Behaviors and Outcomes. Health Behav Res. 2019;2:no pagination.
[26] Juul L, Andersen VJ, Arnoldsen J, Maindal HT. Effectiveness of a brief theory-based health promotion intervention among adults at high risk of type 2 diabetes: One-year results from a randomised trial in a community setting. Primary Care Diabetes. 2016;10:111-20.
[27] Knowler WC, Barrett-Connor E, Fowler SE, Hamman RF, Lachin JM, Walker EA, et al. Reduction in the incidence of type 2 diabetes with lifestyle intervention or metformin. N Engl J Med. 2002;346:393-403.
[28] West DS, Prewitt T, Bursac Z, Felix HC. Weight loss of black, white, and Hispanic men and women in the diabetes prevention program. Obesity. 2008;16:1413-20.
[29] Kramer MK, Molenaar DM, Arena VC, Venditti EM, Meehan RJ, Miller RG, et al. Improving employee health: evaluation of a worksite lifestyle change program to decrease risk factors for diabetes and cardiovascular disease. Journal of occupational and environmental medicine. 2015;57:284-91.
[30] Kramer M, Vanderwood KK, Arena VC, Miller RG, Meehan R, Eaglehouse YL, et al. Evaluation of a diabetes prevention program lifestyle intervention in older adults: A randomized controlled study in three senior/community centers of varying socioeconomic status. The Diabetes Educator. 2018;44:118-29.
[31] Limaye T, Kumaran K, Joglekar C, Bhat D, Kulkarni R, Nanivadekar A, et al. Efficacy of a virtual assistance-based lifestyle intervention in reducing risk factors for Type 2 diabetes in young employees in the information technology industry in India: LIMIT, a randomized controlled trial. Diabetic medicine. 2017;34:563-8.
[32] Moungngern Y, Sanguanthammarong S, Teparak P, Sriwijitkamol A. Effects of a health promotion program conducted by nurses on stabilization of HBA1C in subjects at risk for diabetes: a phase III randomized controlled trial. Chotmaihet thangphaet [journal of the medical association of thailand]. 2018;101:1343-8.
[33] Muralidharan S, Ranjani H, Mohan Anjana R, Jena S, Tandon N, Gupta Y, et al. Engagement and Weight Loss: results from the Mobile Health and Diabetes Trial. Diabetes technology & therapeutics. 2019;21:507-13.

[34] Nanditha A, Thomson H, Susairaj P, Srivanichakorn W, Oliver N, Godsland IF, et al. A pragmatic and scalable strategy using mobile technology to promote sustained lifestyle changes to prevent type 2 diabetes in India and the UK: a randomised controlled trial. Diabetologia. 2020;63:486-96.
[35] Ockene IS, Tellez TL, Rosal MC, Reed GW, Mordes J, Merriam PA, et al. Outcomes of a Latino community-based intervention for the prevention of diabetes: the Lawrence Latino Diabetes Prevention Project. American journal of public health. 2012;102:336-42.
[36] O'Reilly S, Versace V, Mohebbi M, Lim S, Janus E, Dunbar J. The effect of a diabetes prevention program on dietary quality in women with previous gestational diabetes. BMC women's health. 2019;19:88.
[37] O'Reilly SL, Dunbar JA, Versace V, Janus E, Best JD, Carter R, et al. Mothers after Gestational Diabetes in Australia (MAGDA): A Randomised Controlled Trial of a Postnatal Diabetes Prevention Program. PLoS Med. 2016;13:e1002092.
[38] Li G, Zhang P, Wang J, Gregg EW, Yang W, Gong Q, et al. The long-term effect of lifestyle interventions to prevent diabetes in the China Da Qing diabetes prevention study: A 20-year follow-up study. The Lancet. 2008;371:1783-9.
[39] Pan X, Li G, Hu Y. Effect of dietary and/or exercise intervention on incidence of diabetes in 530 subjects with impaired glucose tolerance from 1986-1992. Zhonghua nei ke za zhi [Chinese journal of internal medicine]. 1995;34:108-12.
[40] Pan XR, Li GW, Hu YH, Wang JX, Yang WY, An ZX, et al. Effects of diet and exercise in preventing NIDDM in people with impaired glucose tolerance. The Da Qing IGT and Diabetes Study. Diabetes care. 1997;20:537-44.
[41] Parikh P, Simon EP, Fei K, Looker H, Goytia C, Horowitz CR. Results of a pilot diabetes prevention intervention in East Harlem, New York City: project HEED. American journal of public health. 2010;100 Suppl 1:S232-9.
[42] Patel RM, Misra R, Raj S, Balasubramanyam A. Effectiveness of a Group-Based Culturally Tailored Lifestyle Intervention Program on Changes in Risk Factors for Type 2 Diabetes among Asian Indians in the United States. Journal of diabetes research. 2017;2017:2751980.
[43] Peacock AS, Bogossian FE, Wilkinson SA, Gibbons KS, Kim C, McIntyre HD. A randomised controlled trial to delay or prevent type 2 diabetes after gestational diabetes: walking for exercise and nutrition to prevent diabetes for you. International journal of endocrinology. 2015;2015:423717.
[44] Ramachandran A, Snehalatha C, Mary S, Mukesh B, Bhaskar AD, Vijay V. The Indian Diabetes Prevention Programme shows that lifestyle modification and metformin prevent type 2 diabetes in Asian Indian subjects with impaired glucose tolerance (IDPP-1). Diabetologia. 2006;49:289-97.
[45] Snehalatha C, Mary S, Joshi VV, Ramachandran A. Beneficial effects of strategies for primary prevention of diabetes on cardiovascular risk factors: results of the Indian Diabetes Prevention Programme. Diabetes & vascular disease research. 2008;5:25-9.
[46] Nanditha A, Snehalatha C, Raghavan A, Vinitha R, Satheesh K, Susairaj P, et al. The post-trial analysis of the Indian SMS diabetes prevention study shows persistent beneficial effects of lifestyle intervention. Diabetes research and clinical practice. 2018;142:213-21.
[47] Ram J, Selvam S, Snehalatha C, Nanditha A, Simon M, Shetty AS, et al. Improvement in diet habits, independent of physical activity helps to reduce incident diabetes among prediabetic Asian Indian men. Diabetes research and clinical practice. 2014;106:491-5.
[48] Ramachandran A, Snehalatha C, Ram J, Selvam S, Simon M, Nanditha A, et al. Effectiveness of mobile phone messaging in prevention of type 2 diabetes by lifestyle modification in men in India: a prospective, parallel-group, randomised controlled trial. The lancet Diabetes & endocrinology. 2013;1:191-8.
[49] den Boer AT, Herraets IJ, Stegen J, Roumen C, Corpeleijn E, Schaper NC, et al. Prevention of the metabolic

syndrome in IGT subjects in a lifestyle intervention: results from the SLIM study. Nutrition, metabolism, and cardiovascular diseases : NMCD. 2013;23:1147-53.
[50] Roumen C, Corpeleijn E, Feskens EJ, Mensink M, Saris WH, Blaak EE. Impact of 3-year lifestyle intervention on postprandial glucose metabolism: the SLIM study. Diabetic medicine. 2008;25:597-605.
[51] Roumen C, Feskens EJ, Corpeleijn E, Mensink M, Saris WH, Blaak EE. Predictors of lifestyle intervention outcome and dropout: the SLIM study. European journal of clinical nutrition. 2011;65:1141-7.
[52] Sakane N, Sato J, Tsushita K, Tsujii S, Kotani K, Tominaga M. Effect of baseline HbA1c level on the development of diabetes by lifestyle intervention in primary healthcare settings: insights from subanalysis of the Japan Diabetes Prevention Program. BMJ open diabetes research & care. 2014;2:e000003.
[53] Sakane N, Sato J, Tsushita K, Tsujii S, Kotani K, Tsuzaki K, et al. Prevention of type 2 diabetes in a primary healthcare setting: three-year results of lifestyle intervention in Japanese subjects with impaired glucose tolerance. BMC public health. 2011;11:40.
[54] Shek NW, Ngai CS, Lee CP, Chan JY, Lao TT. Lifestyle modifications in the development of diabetes mellitus and metabolic syndrome in Chinese women who had gestational diabetes mellitus: a randomized interventional trial. Archives of gynecology and obstetrics. 2014;289:319-27.
[55] Lotfaliany M, Sathish T, Shaw J, Thomas E, Tapp RJ, Kapoor N, et al. Effects of a lifestyle intervention on cardiovascular risk among high-risk individuals for diabetes in a low- and middle-income setting: secondary analysis of the Kerala Diabetes Prevention Program. Preventive medicine. 2020:106068.
[56] Thankappan KR, Sathish T, Tapp RJ, Shaw JE, Lotfaliany M, Wolfe R, et al. A peer-support lifestyle intervention for preventing type 2 diabetes in India: a cluster-randomized controlled trial of the Kerala Diabetes Prevention Program. Plos medicine. 2018;15:e1002575.
[57] Van Name MA, Camp AW, Magenheimer EA, Li F, Dziura JD, Montosa A, et al. Effective Translation of an Intensive Lifestyle Intervention for Hispanic Women With Prediabetes in a Community Health Center Setting. Diabetes care. 2016;39:525-31.
[58] Weber MB, Ranjani H, Staimez LR, Anjana RM, Ali MK, Narayan KM, et al. The Stepwise Approach to Diabetes Prevention: results From the D-CLIP Randomized Controlled Trial. Diabetes care. 2016;39:1760-7.
[59] Miller CK, Weinhold KR, Marrero DG, Nagaraja HN, Focht BC. A translational worksite diabetes prevention trial improves psychosocial status, dietary intake, and step counts among employees with prediabetes: a randomized controlled trial. Preventive medicine reports. 2015;2:118-26.
[60] Miller CK, Weinhold KR, Nagaraja HN. Impact of a worksite diabetes prevention intervention on diet quality and social cognitive influences of health behavior: A randomized controlled trial. Journal of Nutrition Education and Behavior. 2016;48:160-9.
[61] Weinhold KR, Miller CK, Marrero DG, Nagaraja HN, Focht BC, Gascon GM. A randomized controlled trial translating the diabetes prevention program to a university worksite, Ohio, 2012-2014. Preventing Chronic Disease. 2015;12:150301.
[62] Wong CK, Fung CS, Siu SC, Lo YY, Wong KW, Fong DY, et al. A short message service (SMS) intervention to prevent diabetes in Chinese professional drivers with pre-diabetes: a pilot single-blinded randomized controlled trial. Diabetes research and clinical practice. 2013;102:158-66.
[63] Yates T, Edwardson CL, Henson J, Gray LJ, Ashra NB, Troughton J, et al. Walking Away from Type 2 diabetes: a cluster randomized controlled trial. Diabetic medicine. 2017;34:698-707.
[64] Zilberman-Kravits D, Meyerstein N, Abu-Rabia Y, Wiznitzer A, Harman-Boehm I. The impact of a cultural lifestyle intervention on metabolic parameters after gestational diabetes mellitus a randomized controlled trial. Maternal and Child Health Journal. 2018;22:803-11.
